# Prevalence and association between alcohol, tobacco, and COVID-19: a study from a tribal predominant district in eastern India

**DOI:** 10.3389/fpubh.2024.1415178

**Published:** 2024-08-16

**Authors:** Venkata Lakshmi Narasimha, Santanu Nath, Benazir Alam, Bipasa Kumari, Pooja Kumari, Shalini Kumari, Jagdish Kaur, Saurabh Varshney

**Affiliations:** ^1^National Institute of Mental Health and Neurosciences, Bangalore, India; ^2^All India Institute of Medical Sciences, Deoghar, India; ^3^World Health Organization-South-East Asia Regional Office, New Delhi, India

**Keywords:** tobacco, alcohol, COVID-19, India, addiction, pandemic

## Abstract

**Introduction:**

Alcohol and tobacco use has been proposed to significantly affect COVID-19 outcomes. The study aimed to estimate the prevalence of alcohol and tobacco use among COVID-19-positive patients and compare it with the general population prevalence rates. It also aimed to assess and determine the association between the severity of COVID-19 illness and the complications with alcohol and tobacco use.

**Method:**

For this, a cross-sectional, retrospective, telephone-based study was conducted using a structured questionnaire among COVID-19 diagnosed patients in the district of Deoghar of the Indian state of Jharkhand. A multinomial logistic regression is done to determine the association.

**Results:**

Among 1,425 patients interviewed, tobacco and alcohol were used by 22.31 and 9.96%, significantly more than the prevalence of tobacco (*Z* = 4.9485, *p* < 0.00001) and alcohol use (*Z* = 7.118, *p* < 0.00001), respectively, in the district (tobacco-11.7% and alcohol-4.8%).

In a regression model, patients with co-morbidity had higher odds of severe [3.34 (1.99–5.62)] and moderate [2.95 (1.97–4.41)] COVID-19. Young [0.12 (0.04–0.38)] and middle-aged individuals [0.23 (0.13–0.4)], people below the poverty line 0.28 (0.11–0.69) are at lower odds of severe COVID-19. Tobacco users [1.58 (1.16–2.14)], alcohol users [1.53 (1.03–2.28)], incomplete vaccination [3.24 (1.49–7.01)], and patients with comorbidity [3.6 (2.79–4.68)] were found to have higher odds of post-COVID-19 complications.

**Discussion:**

People with COVID-19 in our study population had significantly higher tobacco and alcohol use compared to the general population. Tobacco and alcohol use significantly increases the risk of post-COVID-19 complications. The study highlights the need for addiction treatment services to prevent complications during future pandemics.

## Introduction

In India, a quarter of the population uses tobacco (11% in the Deoghar district), and around 5% suffer from alcohol use disorder (1). The COVID-19 pandemic has significantly affected the Indian population. People in isolation or with burgeoning mental health issues due to the pandemic remained at an increased risk of using psychoactive substances, tobacco, and alcohol being the commonest of them (2, 3). Tobacco use, mainly in smoking, is an established risk factor for several diseases, including cardiovascular and chronic lung diseases. It is known that this can increase susceptibility to viral respiratory infections, including COVID-19 (4, 5). Smoking has been shown to increase the risk of hospitalization [relative risk (RR)-1.2 (1.03–1.44)], the severity of disease [RR-1.52 (1.13–2.07)] and mortality [RR-1.39 (1.09–1.87)] due to COVID-19 (6). In a recent systematic review to find an association between smoking and COVID-19, the authors concluded that “smoking is most likely associated with negative progression and adverse outcomes of COVID-19” (7). The same is also true for alcohol. People with AUDs are known to be vulnerable to acute lung injury and acute respiratory distress syndrome and have poorer cardiac outcomes (8). This is possible through alcohol-induced endothelial injury, ciliary dysfunction, alteration of glutathione homeostasis etc. (9). COVID-19 being a predominantly respiratory infection, with its effect on cardiac and other essential body systems, alcohol is likely to bring deleterious outcomes in those affected by COVID-19.

Although preliminary evidence suggests alcohol and tobacco use adversely affects the course of COVID-19 illness yet, there is less data to establish such an association.

Our study aims to systematically assess the prevalence and effects of alcohol and tobacco use among COVID-19 patients. The primary objective is to estimate the prevalence of alcohol and tobacco use among COVID-19-positive patients in the Deoghar district, Jharkhand state, India, and compare it with the general population prevalence of the Deoghar district. The secondary objective is to assess and determine the association between the severity of COVID-19 illness and the complications with alcohol and tobacco use.

## Materials and methods

### Study design, setting, and population

The study was a telephonic-based cross-sectional survey involving patients from the Deoghar district of Jharkhand, which falls under the Santhal Parganas, a tribal predominant state in the Eastern part of India. This district was selected for the index study because it falls under the immediate catchment area of the tertiary medical institute from where this index study was conducted. Secondly, administrative approval to obtain COVID-19 related patient data in this district was readily obtained, considering this institute’s strategic location at the district headquarters in Deoghar. The study included people who provided informed consent, above 12 years of age, of both gender and residing in the district of Deoghar, who tested positive for COVID-19 (either with RT-PCR/Rapid antigen) during the second wave of the pandemic (from 1st February 2021 to 31st October 2021, i.e., 9 months), and has been recorded in the national portal of India. Participants who were positive during the telephonic data collection were excluded from the study. The information was collected from the patient. Family members were interviewed if the patient was unavailable for the interview or died during the COVID-19 infection.

### Sample size and sampling design

The sample was drawn from the study population by simple random sampling from a list of COVID-19 patients. The list was obtained with permission from the district COVID-19 management cell, Department of Health and Family Welfare, Government of Jharkhand. A sample size of 1,413 was based on the study’s objectives (see Supplementary material).

### Study tools and questionnaire

Counselors did a telephone-based survey using a structured questionnaire administered orally after consent. On average, the interview lasted for 10–12 min. The questionnaire (see Supplementary material) consisted of details related to socio-demographic data, the severity of COVID-19 disease as ascertained and framed based on the Indian Council of Medical Research (ICMR) gradation of mild, moderate and severe disease during the second wave of COVID-19 pandemic (10). The question related to complications due to COVID-19 was framed based on Nalbandian et al. (11) post-acute COVID syndrome. The questions (10 in total) included commonly encountered cardiovascular, hematological, and neurological complications. Mental health complications were assessed using the following screening tools:

- Patient health questionnaire-2 (PHQ-2) (12)
- Generalized anxiety disorder-2 (GAD-2) (13)
- Primary care PTSD screen for DSM-5 (PTSD-5) (14)

The severity of tobacco and alcohol use was ascertained using the following tools:

- Fagerstorm test for nicotine dependence (FTND) (15)
- Fagerstorm test for nicotine dependence-smokeless tobacco (FTND-ST) (16)
  The FTND and FTND-ST provide scores for the use severity based on the type of tobacco/nicotine (smoked/smokeless). Based on the scores obtained, mild, moderate and severe dependence on tobacco/nicotine were used.
- Alcohol use disorder identification test (AUDIT) (17)

All the scales used for the study are validated in the Indian population. Ethical approval was taken from the institute ethics committee (2022-35-EMP-02). Detailed data collection training and procedure explained in the Supplementary material.

### Statistical analysis

All the data was collected using HIPPA-compliant software (SurveyMonkey). The data were analyzed using Excel and R software (18). Variables (sociodemographic and clinical parameters) were subjected to descriptive analysis using frequencies and percentages. Chi-square and multinominal logistic regression were used for association. A structural equation modeling was attempted using the lavaan package in R (19).

## Results

### Socio-demographic profile

Among 3,018 people contacted, 1,425 subjects were interviewed telephonically during the period, as mentioned before. Most of the COVID-19 sufferers included in the study were males (*n* = 995, 70%) and were in an urban location (*n* = 1,117, 78%). They were mainly of the age group of 30–60 years (middle age) (*n* = 1,016, 71%). Regarding socio-economic status, 26% of the participants/responders were below the poverty level (BPL). A meager 7% of the sufferers (*n* = 105) interviewed worked in a hospital or were associated with one when they got infected (Table 1).

**Table 1:** Socio-demographic and clinical profile of study participants.

S.no	Variable	*N* (Total = 1,425) (%)
Respondent		
1	Patient	766 (54%)
2	Family member for the patient (patient alive)	639 (45%)
3	Family member for the patient (patient dead)	20 (1%)
Gender		
1	Male	995 (70%)
2	Female	430 (30%)
Age (in years)		
1	Young age (< 30 years)	251 (18%)
2	Middle age (31–60 Years)	1,016 (71%)
3	Older adult (> 60 years)	158 (11%)
Socioeconomic status (SES)		
1	APL card holder	1,052 (74%)
2	BPL card holder	373 (26%)
Occupation		
1	Neither home nor hospital based/related work	1,185 (83%)
2	Work from/at home	135 (9%)
3	Work in/associated with hospital	105 (7%)
Background		
1	Urban	1,117 (78%)
2	Rural	308 (22%)
Severity		
1	Mild	1,222 (86%)
2	Moderate	125 (9%)
3	Severe	78 (5%)
Vaccination		
1	Not completed vaccination	1,356 (95%)
2	Completed vaccination (Two doses)	69 (5%)
Co-morbidities		
1	Diabetes	197 (13.8%)
2	Hypertension	253 (17.7%)
3	Heart disease	46 (3.2%)
4	Kidney disease	14 (0.9%)
5	Cancer	6 (0.4%)
6	Chronic lung disease	18 (1.2%)
7	Mental illness	17 (1.1%)
8	Other diseases	162 (11.2%)
Post COVID-19 complications		
1	Heart disease	47 (3.2%)
2	Kidney disease	15 (1%)
3	Lung disease	55 (3.8%)
4	Blood disease	41 (2.8%)
5	Auto-immune disease	86 (6%)
6	New infections	73 (5.2%)
7	Neurological problems	15 (1%)
8	Anosmia	150 (11%)
9	Agnosia	25 (1.7%)
10	Psychological problems	127 (9%)
11	Others	135 (9.4%)

APL, Above poverty line; BPL, Below poverty line.

### Clinical profile of the sample

While most of the interviewed patients had a mild illness (*n* = 1,222, 86%), 5% (*n* = 78) had severe COVID-19. Only 5% (*n* = 69) had received both doses of COVID-19 vaccination when they got infected. Among the co-morbidities assessed, the majority of the COVID-19 sufferers had hypertension (*n* = 253, 17.7%) and Diabetes Mellitus (DM) (*n* = 197, 13.8%), mental illness was comorbid in 1.1% (*n* = 17) of the studied sample (Table 1). A total 11.2% (*n* = 162) of the studied population had other comorbidities.

### COVID-19-related medical complications

The study samples reported various physical/medical complications following their COVID-19 infection. Anosmia was the most common post-COVID-19 complication in the studied sample (*n* = 150, 11%) (Table 1).

### Post COVID-19 psychological complications

Psychological problems were recognized as complications in 9% (*n* = 127) of them. 118 (8.3%) participants were screened to have a post-COVID psychiatric illness (see Supplementary material). Psychological constructs which were screened were anxiety, depression and PTSD. While anxiety was screened positive in 124 (8.7%) study participants using the GAD-2 scale, 108 (7.6%) screened positive for depression assessed using the 2-item PHQ-2 questionnaire. The proportion of the study population who reported having experienced PTSD symptoms, as screened by the PTSD-5 questionnaire, was, however, not less (*n* = 100, 7%).

### Tobacco before and after COVID-19 infection

Of all the samples of patients (*n* = 1,425), 318 (22.31%) used tobacco. The majority of the users consumed smokeless form (e.g., Gutka, khaini, other chewable products), 16% (*n* = 232) and 5.5% (*n* = 79) smoked tobacco, while a few used both (*n* = 7, 0.5%). Males with COVID-19 comprised the majority of both the smoked (*n* = 76, 96%) and smokeless tobacco (SMT) (*n* = 225, 97%) users. The current tobacco users (used within 3 months before the COVID-19 infection) were 252 (79.2%). Among smokeless and smoking, 203 (88%) and 42 (53%) were current users. Patterns and severity of tobacco consumption can be understood from the Supplementary material.

### Alcohol use before and after COVID-19 infection

Regarding alcohol use, 9.2% (*n* = 132) were users within 3 months of testing positive, while 0.7% (*n* = 10) were not users within 3 months before testing positive. Among those who consumed alcohol, the majority of them were males (*n* = 121, 85%).

### Comparison of prevalence rates between the study population and general population prevalence rates

**Figure 1:**
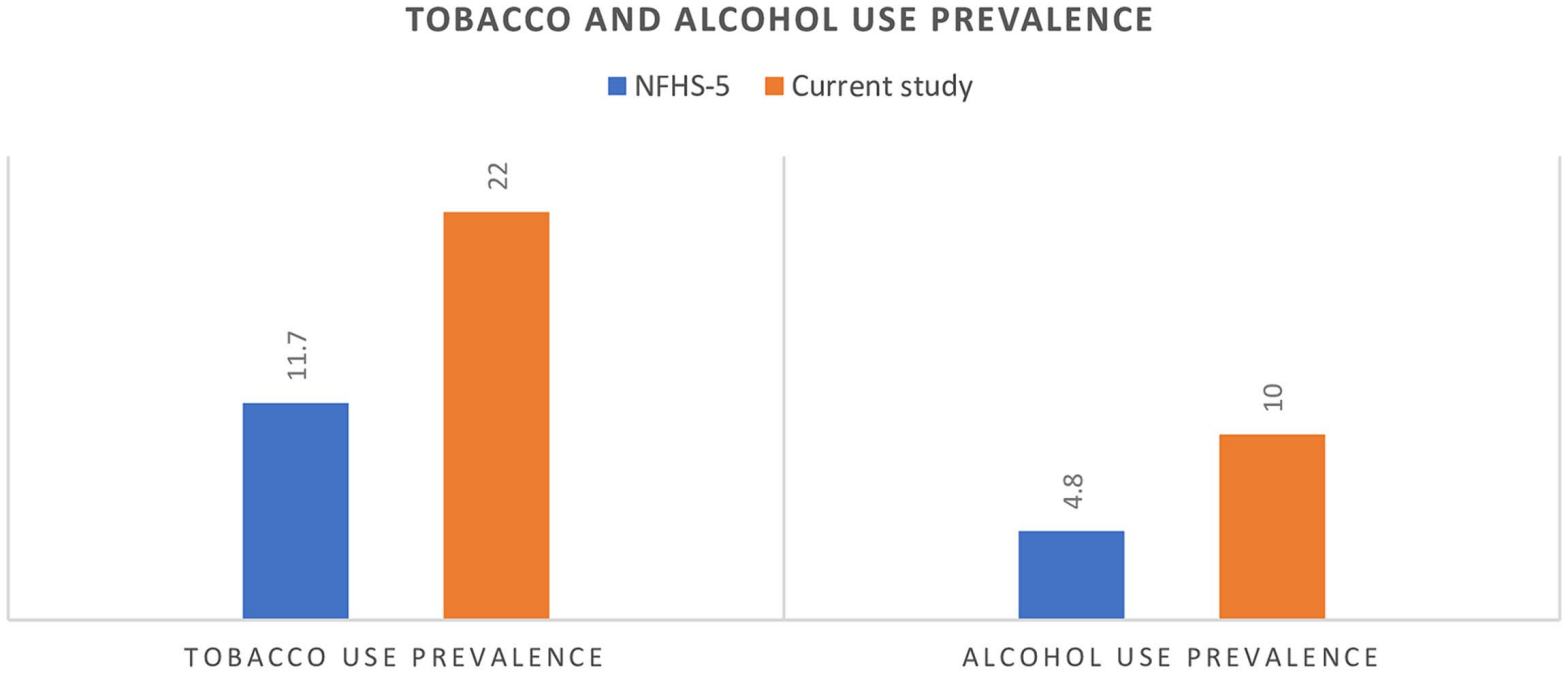
National family health survey-5 (NFHS-5) vs. current study finding of tobacco and alcohol use prevalence.

Prevalence of tobacco and alcohol use in the study population was compared with the prevalence rates of the population of Deoghar district by NFHS-5 data: in a two-sample proportion *Z*-test, compared to NFHS-5 (4.83%), the prevalence of alcohol use is higher in our study (9.96%) (*Z* = 7.118, *p* < 0.00001). Similarly, compared to NFHS-5 (11.74%), the prevalence of Tobacco use is higher in our study (22%) (*Z* = 4.9485, *p* < 0.00001) (Figure 1).

### Bivariate association between tobacco use, alcohol use, and COVID-19 severity, complications

The results of bivariate association were described for tobacco and alcohol use; for the rest of the variables, please see the Supplementary material. A significantly greater proportion of tobacco users had moderate COVID-19 infection compared to non-users (*p* = 0.03) (Table 2). Both tobacco users (*p* < 0.01) and alcohol (*p* < 0.01) users had greater complications compared to non-users (Table 3).

**Table 2:** Association between tobacco use, alcohol use, and COVID-19 severity.

S.no	Independent variables	Severity			Chi-square	Degree of freedom	*p*-value
		Mild	Moderate	Severe			
1.	Tobacco use:	83.01%	**12.26%**	4.71%	6.4766	2	**0.03**
	Yes						
	No	86.54%	**7.76%**	5.69%			
2.	Alcohol use:	85.21%	9.15%	5.63%	0.0391	2	0.98
	Yes						
	No	85.81%	8.72%	5.45%			

Values in bold are statistically signgificant.

**Table 3:** Association between tobacco use, alcohol use, and COVID-19 complications.

S.no	Independent variables	Complications		Chi-square	Degree of freedom	*p*-value
		Present	Absent			
1.	Tobacco use:	38.99%	61.00%	20.233	1	**<0.0001**
	Yes					
	No	25.83%	74.16%			
2.	Alcohol use:	40.14%	59.85%	9.3404	1	**<0.002**
	Yes					
	No	27.51%	72.48%			

Values in bold are statistically signgificant.

### Multinomial logistic regression model

We employed a multinomial logistic regression model to understand the association between different independent variables and COVID-19 illness severity and complications as dependent variables. The dependent variable, COVID-19 severity, was categorized into three levels: mild, Moderate, and Severe, and for COVID-19 complications as present or absent. The independent variables included tobacco use (yes or no), alcohol use (yes or no), age group (older adult, middle age, young), gender (male, female), background (urban, rural), socio-economic status (above or below poverty line), occupation (work from home, work in/associated with hospital or neither), age group, presence of co-morbidity (yes or no), vaccination status (complete or incomplete). All the independent variables were known to have affected the severity of COVID-19 and its complications. For a detailed analysis, see RPubs-Alcohol Tobacco and COVID-19 (20).

### Association between socio-demographic and clinical variables with severity

Table 4 represents the association between moderate COVID-19 severity and the independent variables using multinomial logistic regression analysis. Tobacco users had higher odds of 1.40 (0.89–2.19, *p* = 0.13) moderate COVID-19 severity. However, the association was not statistically significant. Similarly, alcohol use (OR = 0.91 (0.48–1.72), *p* = 0.79) and gender (OR = 0.9 (0.58–1.42), *p* = 0.67) did not show statistically significant associations with the outcome. On the other hand, individuals who reported working from/at home had an odds ratio of 1.76 (1.003–3.10). This association was statistically significant (*p* = 0.048). Middle age group is associated with lower odds of severity 0.5852 (0.34–0.98, *p* = 0.04). The presence of a co-morbidity [OR = 2.95 (1.97–4.41), *p* < 0.0001] demonstrated a strong and statistically significant association with moderate severity, suggesting higher odds of the outcome among individuals with a co-morbidity. Among the examined factors, the association between tobacco use and severe COVID-19 was not statistically significant (*p* = 0.1828), with an odds ratio of 0.6479 (0.34–1.22) (Table 5). On the other hand, individuals in the middle age group had significantly (*p* < 0.0001) lower odds of 0.23 (0.13–0.40) severe COVID-19 infection compared to the older adult. Similarly, individuals in the young age group had a lower odds ratio of 0.12 (0.04–0.38). This association was also statistically significant (*p* = 0.0003).

**Table 4:** Coefficients and odds ratios of multinomial logistic regression analysis for COVID-19 severity (moderate).

	Coefficient	Std. errors	OR	95% CI	z stat	*p*-value
(Intercept)	−2.1776	0.6177	0.1133	[0.0338, 0.3803]	−3.5251	0.0004
Tobacco use	0.3385	0.2291	1.4028	[0.8953, 2.1980]	1.4774	0.1396
Alcohol use	−0.0858	0.3221	0.9178	[0.4882, 1.7254]	−0.2663	0.7900
Gender: Male	−0.0948	0.2292	0.9095	[0.5804, 1.4254]	−0.4137	0.6791
Occupation: work from/at home	0.5686	0.2886	1.7658	[1.0030, 3.1089]	1.9702	**0.0488**
Occupation: work in/associated with a hospital	0.2013	0.3745	1.2230	[0.5870, 2.5478]	0.5375	0.5909
Background: urban	−0.3357	0.2757	0.7148	[0.4164, 1.2270]	−1.2177	0.2233
Vaccination: not completed	0.1792	0.4990	1.1962	[0.4499, 3.1808]	0.3591	0.7195
Middle age	−0.5357	0.2667	0.5852	[0.3470, 0.9870]	−2.0090	**0.0445**
Young age	−0.5713	0.3719	0.5648	[0.2725, 1.1707]	−1.5362	0.1245
Co-morbidity	1.0830	0.2048	2.9535	[1.9769, 4.4124]	5.2876	**0.0000**
BPL card holder	−0.5318	0.2791	0.5875	[0.3400, 1.0152]	−1.9058	0.0567

BPL, Below poverty line. Values in bold are statistically signgificant.

**Table 5:** Coefficients and odds ratios of multinomial logistic regression analysis for COVID-19 severity (severe).

	Coefficient	Std. Errors	OR	95% CI	z stat	*p*-value
(Intercept)	−2.1367	0.8773	0.1180	[0.0211, 0.6589]	−2.4354	0.0149
Tobacco use	−0.4340	0.3257	0.6479	[0.3422, 1.2268]	−1.3323	0.1828
Gender: male	0.0368	0.2813	1.0375	[0.5978, 1.8006]	0.1307	0.8960
Occupation: work from/at home	−0.3460	0.4937	0.7075	[0.2689, 1.8619]	−0.7009	0.4834
Occupation: work in/associated with a hospital	−0.4968	0.6259	0.6085	[0.1784, 2.0752]	−0.7937	0.4274
Background: urban	−0.0533	0.3921	0.9481	[0.4396, 2.0446]	−0.1359	0.8919
Vaccination: not completed	0.3887	0.7606	1.4751	[0.3322, 6.5498]	0.5111	0.6093
Alcohol use	0.1479	0.4050	1.1594	[0.5242, 2.5645]	0.3653	0.7149
Middle age	−1.4487	0.2768	0.2349	[0.1365, 0.4041]	−5.2339	**0.0000**
Young age	−2.0789	0.5733	0.1251	[0.0407, 0.3847]	−3.6265	**0.0003**
Co-morbidity	1.2078	0.2646	3.3461	[1.9923, 5.6200]	4.5653	**0.0000**
BPL card holder	−1.2616	0.4563	0.2832	[0.1158, 0.6927]	−2.7647	**0.0057**

BPL, Below poverty line. Values in bold are statistically signgificant.

Furthermore, co-morbidity was strongly associated with severe COVID-19, odds ratio of 3.34 (1.99–5.62). This association was statistically significant (*p* < 0.0001). Additionally, individuals who were BPL (Below Poverty Line) cardholders had lower odds of 0.28 (0.11–0.69) of severe COVID-19 infection. This association was statistically significant (*p* = 0.0057).

### Post-COVID-19 complications

Table 6 represents the association between post-COVID-19 complications (presence) and the independent variables using multinomial logistic regression analysis. Among the examined factors, several variables showed statistically significant associations with post-COVID-19 complications. Specifically, tobacco users had higher odds of post-COVID-19 complications at 1.58 (1.16–2.14). This association was statistically significant (*p* = 0.0033). Alcohol Use was also significantly associated with post-complications, with an odds ratio of 1.53 (1.03–2.28) (*p* = 0.0315). Individuals working in or associated with hospitals and incompletely vaccinated had higher odds of post-COVID-19 complications. People below the poverty line have lower odds of post-COVID-19 complications (Table 6). Other variables, including Gender (Male), Background (Urban), Middle Age, and Young Age, did not show statistically significant associations with post-COVID-19 complications (*p* > 0.05).

**Table 6:** Coefficients and odds ratios of multinomial logistic regression analysis for post-COVID-19 complications (presence) with independent variables.

Variable	Coefficient	Std. errors	OR	95% CI	z stat	*p*-value
(Intercept)	−2.7626591	0.4741675	0.0631	0.0249–0.1599	−5.8263354	0.0000000
Tobacco use	0.4584430	0.1560563	**1.5816**	**1.1648–2.1475**	2.9376781	**0.0033068**
Alcohol use	0.4315920	0.2007126	**1.5397**	**1.0389–2.2818**	2.1502991	**0.0315316**
Gender: male	−0.0046343	0.1514233	0.9954	0.7398–1.3393	−0.0306046	0.9755848
Occupation: work from/at home	0.4878464	0.2163415	1.6288	1.0659–2.4890	2.2549829	**0.0241344**
Occupation: work in/associated with a hospital	0.6516429	0.2394029	1.9187	1.2001–3.0675	2.7219508	**0.0064898**
Background: urban	0.2042602	0.1948345	1.2266	0.8373–1.7970	1.0483781	0.2944645
Vaccination: not completed	1.1757548	0.3937252	3.2406	1.4979–7.0107	2.9862320	**0.0028244**
Middle age	−0.0366556	0.1952475	0.9640	0.6575–1.4134	−0.1877394	0.8510810
Young age	−0.3840448	0.2606596	0.6811	0.4086–1.1352	−1.4733574	0.1406547
Comorbidity	1.2861848	0.1315558	3.6190	2.7964–4.6834	9.7767280	**<0.001**
BPL card holder	−0.7038594	0.1842346	0.4947	0.3447–0.7098	−3.8204517	**0.0001332**

BPL, Below poverty line. Values in bold are statistically signgificant.

## Discussion

### The main findings of this study

The study assessed the prevalence of tobacco and alcohol use among patients who suffered from COVID-19 during the second wave of the pandemic and their effect on COVID-19 outcomes, the severity of the infection and post-COVID-19 complications. The prevalence of alcohol and tobacco use among patients with COVID-19 illness in Deoghar Jharkhand is higher than the general population prevalence rates. Tobacco use, alcohol use, incomplete vaccination, and patients with co-morbidity had higher odds of post-COVID-19 complications. While patients with co-morbidity had higher odds of COVID-19 severity, young age and individuals from below the poverty line had lower odds of COVID-19 severity.

### What was already known on the topic

Tobacco use, specifically smoking (current and former), increases the risk of COVID-19 progression (by 30–50%) and mortality (21, 22). Similarly, alcohol use is associated with higher COVID-19 severity (23). It is currently unclear how tobacco and alcohol consumption may affect post COVID-19 complications. In our study, the majority of participants used chewable tobacco rather than smoking.

### What this study adds

#### Co-morbidity, COVID-19 severity, and post COVID-19 complications

Hypertension (17.7%) and diabetes (13.8%) were the most common comorbidities in our study population, similar to other studies conducted in India (24). Epidemiological research suggests that most people suffer from mild COVID-19 illness (25). In our study, 86% had a mild illness. New onset health complications following COVID-19 infection, apart from its primary effect on the respiratory system, impacted recovered patients’ morbidity and quality of life. Anosmia was the most common complication, followed by psychological complications (anxiety and depression). The incidence of anosmia was reported to be ranging widely from 9.2 to 82% in different Indian studies (26). The disparity in the prevalence of psychological complications between our findings (lower prevalence) and those of the rest (higher prevalence) can be explained by the fact that there could have been a recall bias in our study, which might have accentuated a telephonic interview (27, 28). Secondly, the stigma toward mental illness could have led to lesser reporting. Third, verbalizing mental health-related symptoms to an invisible interviewer over the telephone can be contributory.

### Tobacco and alcohol use

Tobacco and alcohol were used by 22% and 10% of the study population. Compared to most research worldwide, in our study, most tobacco users consumed chewable tobacco (21). Hence, these findings are different from the other studies. Similar to GATS-2 data, our population were predominantly tobacco chewers.

These prevalence rates were significantly higher than the national surveys (NFHS-5) prevalence rates of the Deoghar district. First, the NFHS-5 study population in the district were predominantly women. Secondly, tobacco and alcohol prevalence was one of the objectives of the national survey. Here, it is our primary objective.

### Association of tobacco and alcohol use with COVID-19 severity and post COVID-19 complications

Individuals with co-morbidity have a higher severity of COVID-19 infection. At the same time, the young population and those below the poverty line are at lower odds of severe COVID-19 illness. The bivariate analysis highlighted that tobacco use increased the severity of COVID-19 infection but failed to achieve significance in regression. The findings are consistent with recent studies in India and other nations. Our study consisted of predominantly smokeless tobacco users. It could be one of the reasons for a non-significant association compared to the previous studies. Our research has shown that the previously held belief that tobacco users are less susceptible to COVID-19 infection is inaccurate. The link between tobacco use and the severity of respiratory infections (including COVID-19) has been discussed in scientific literature along with putative mechanisms for such, which leaves no doubt regarding their association (5).

Though evidence suggests that alcohol use increases the risk of symptomatic infection, we could not establish such an association (29). This could be because the survey was conducted over the phone, which may have resulted in unreported cases. Vaccination status did not determine the illness severity. One possible reason is that only a small percentage (5%) of individuals had received both vaccine doses. During the study period, India was in the early phases of vaccination. Healthcare workers and the older adult were given the first vaccination dose, with a gap for the second dose. This is important as both are at a higher risk of COVID-19 vulnerability, severity and consequent complications. The findings of the vaccine studies that are prospective, long-term and with robust study design undoubtedly confirmed the effectiveness of the vaccination (30, 31).

Tobacco and alcohol users, incomplete vaccination, people with comorbidities, and occupation (work from home and hospital) were found to have higher odds of post-COVID complications. People below the poverty line have lower odds of post-COVID-19 complications.

Our study is among the limited research that investigates the link between tobacco and alcohol use and post COVID-19 complications. Recent evidence suggests that alcohol and tobacco use is associated with adverse outcomes among patients diagnosed with COVID-19 (2, 32, 33). A community-based study conducted on patients in Karnataka, India, revealed that the risks of COVID-19 complications were higher in individuals with advanced age, comorbidities, tobacco, and alcohol consumption (34). In contrast, a systematic review discovered that individuals with a lower socioeconomic status were more prone to contracting COVID-19 and experienced worse outcomes and complications (35). In a recent meta-analysis, vaccinated individuals had a 29% lower risk of developing long COVID than those unvaccinated individuals (36).

Finally, the study results emphasize that tobacco and alcohol consumption continue to add to the disease burden by increasing the likelihood of post-COVID-19 complications. In the background of a higher treatment gap for substance use disorders, there should be a concerted effort from different stakeholders to improve the accessibility and availability of treatment services for quitting tobacco and alcohol. Since they significantly increase complications arising from any communicable and non-communicable diseases (NCDs). Primary care physicians and specialists should provide screening and brief interventions for substance use disorders to help people quit (37). Efficient implementation of drug policies and laws is crucial for minimizing the adverse effects of substance use disorders (38). Further, during pandemics like COVID-19, there should be stronger promulgation of tobacco and alcohol cessation activities from the public health policy perspective and a medical model in effectively managing this substance use problem. In the present time, it is imperative to adopt a comprehensive approach.

The study is not without limitations. Being a retrospective study, there could be a recall bias. As the study was telephone-based, many of the findings are based on self-reporting. There can be underreporting of stigma-related issues, like alcohol and tobacco use. There can be overreporting and a lack of confirmation for COVID-19 severity and complications. Also, this study was conducted in a particular district of a state from eastern India, so the findings cannot be generalized to eastern India or the country at large.

## Conclusion

Tobacco and alcohol use among the COVID-19 population of Deoghar is significantly higher than the general population prevalence rates. Tobacco and alcohol use significantly increases the risk of post-COVID-19 complications. While patients with comorbidity have a higher risk of post-COVID-19 complications, younger age has a lesser risk of complications. The study highlights the need for addiction treatment services to prevent complications during future pandemics.

## Data Availability

The original contributions presented in the study are included in the article/Supplementary material, further inquiries can be directed to the corresponding author.
