# Supplementary material for: Prevalence and association between alcohol, tobacco, and COVID-19: a study from a tribal predominant district in eastern India
Source: Front Public Health. 2024 Aug 16;12:1415178. doi: 10.3389/fpubh.2024.1415178 (PMC11361974; doi:10.3389/fpubh.2024.1415178)

# **Supplementary materials:**

## **Methods:**

### **Sample Size calculation:**

The prevalence of tobacco use in the Deoghar district, according to National Family Health Survey 5 (NFHS-5) 2021 (*NFHS-5_Phase-II_0.Pdf*, n.d.). Using the formula mentioned below with a 95% confidence interval (C.I.) and 80% power with an expected prevalence of 35% in patients with COVID-19, the sample size was calculated to be 1284. Considering a 10% non-response rate, the final sample size was estimated to be 1413.

n = (Zα/2+Zβ)^2^ * (p1(1-p1) + p2(1-p2)) / (p1-p2)^2^

For the secondary objective, tobacco users have a higher risk severity of illness with an OR of 1.5 compared to non-users, with a power of 80% and 95% C.I. The sample size was calculated using online software (https://www.openepi.com). The sample size was calculated as 852. The calculated sample size for the primary objective was larger than the secondary objective, and thus the final sample size for the study was 1413.

### **Training and conduct of study:**

During the initial part of the project, all the staff underwent training for 1week related to the objectives and using the questionnaire. The first 50 calls and after that, five random calls for every 100 calls were supervised by the principal and co-investigators. More than 200 calls were recorded using HIPPA-compliant software (Exotel). Five experts in the field randomly reviewed the 50 recorded calls for the process of the interview. The review included checking for the quality of the introduction, consent process, appropriateness of questions asked, completeness of the questionnaire, language compatibility and ending the interview process. Feedback was provided to the research personnel following each session. The study participants' data collection method is mentioned in Figure 1.

Supplementary Figure 1: Data collection flowchart

## **Results:**

Supplementary Table 1: Responses to the calls during the study.

| **S.no** |  | **No. of calls** |
| --- | --- | --- |
|  | Completed interviews | **1425 (47.2%)** |
|  | Switched off/Not reachable/Not responded/ Out of service/ No incoming/ Does not exist (3 attempts) | 955 |
|  | Not willing to participate in study | 20 |
|  | Hospital staff number | 4 |
|  | Invalid Number/ Wrong number | 610 |
|  | Minor age (<12 years) | 4 |
|  | **Total calls** | **3018** |

Supplementary Table 2: Tobacco use among the study population.

| **S. No** | **Tobacco** | **N (Total=1425) (%)** |
| --- | --- | --- |
|  | Smokeless (such as gutka, khaini, other chewing tobacco) | 232 (16%) |
|  | Smoking (such as bidi, cigarette, cigar, etc) | 79 (5.5%) |
|  | Both | 7 (0.5%) |
|  | Total | 318 (22%) |

Supplementary Table 3: New-onset psychological complications following COVID-19

| **S.no** | **Variable** | **N (Total=1425) (%)** |
| --- | --- | --- |
| **Psychiatric illness** | | |
| 1 | No | 1371 (96%) |
| 2 | Yes | 54 (4%) |
| **GAD-2 Score** | | |
| 1 | No (less than 3) | 1301 (91.2%) |
| 2 | Yes (more than 3 or equal to 3) | 124 (8.7%) |
| **PHQ-2 Score** | | |
| 1 | No (less than 3) | 1317 (92.4%) |
| 2 | Yes (more than 3 or equal to 3) | 108 (7.6%) |
| **PTSD Score** | | |
| 1 | No (0-2 score) | 1325 (93%) |
| 2 | Yes (more than 3 or equal to 3) | 100 (7%) |

The consumption of tobacco and alcohol among the sample was studied in terms of whether they were used within three months before testing COVID-19 positive or not, i.e. not used substances within three months before testing positive (Table 6).

The severity of tobacco and alcohol use before being infected with COVID-19 in the study population was assessed using the FTND (for smoked tobacco), FTND-ST (for smokeless tobacco) and AUDIT (for alcohol) (Table 7). Among smokers (n=79), the majority, 42 (53%), had moderate nicotine dependence severity. The severity pattern for smokeless tobacco is also similar to that of smokers; the majority (n=99, 42.6%) of the smokeless tobacco users were moderately dependent, 73 (31.4%). Among those who smoked tobacco (n=79), the majority (n=52, 66%) continued smoking daily, which outnumbered once weekly use (n=15, 19%) and once monthly use (n=3, 4%). 9 (11%) of them completely abstained from smoking after they got infected with the virus. For SMT users, the picture is nearly the same. While the majority of them (n=193, 83%) chewed tobacco in a daily pattern, a minority of them used SMT in a once monthly (n=22, 9.4%) and once weekly pattern (n=13, 5.6%), while 4 (2%) of the SMT users completely stopped SMT.

Supplementary Table 4: Gender distribution of Tobacco use among the study population.

| **S.no** | **Category** | **Smokeless Tobacco** | **Smoking Tobacco** | **Both** |
| --- | --- | --- | --- | --- |
| 1 | Female | 7 (3%) | 3 (4%) | 0 |
| 2 | Male | 225 (97%) | 76 (96%) | 7 (100%) |
|  | Total | 232 (100%) | 79 (100%) | 7 (100%) |

Supplementary Table 5: Tobacco use within 3 months before the COVID-19 infection

| **S.no** | **Category** | **Smokeless Tobacco** | | **Smoking Tobacco** | | **Both** | |
| --- | --- | --- | --- | --- | --- | --- | --- |
|  |  | **Use within 3 months** | **Not used within 3 months** | **Use within 3 months** | **Not used within 3 months** | **Use within 3 months** | **Not used within 3 months** |
| 1 | Female | 3 (1%) | 4 (2%) | 1(1%) | 2 (3%) | 0 | 0 |
| 2 | Male | 202 (87%) | 23 (10%) | 41(52%) | 35 (44%) | 7 (100%) | 0 |
|  | Total | 232 | | 79 | | 7 | |

Supplementary Table 6: AUDIT, Fagerstrom smoking and smokeless severity scores

| **S.no** | **Category** | **Fagerstrom score** | | | | **AUDIT score** |
| --- | --- | --- | --- | --- | --- | --- |
|  |  | **Smokeless** | **Smoking** | **Both** | |  |
|  |  |  |  | **Smokeless** | **Smoking** |  |
| 1 | Mild | 60 (26%) | 11 (14%) | 2 (29%) | 2 (29%) | 24 (17%) |
| 2 | Moderate | 99 (42.6%) | 42 (53%) | 1 (14%) | 1 (14%) | 40 (28%) |
| 3 | Severe | 73 (31.4%) | 26 (33%) | 4 (57%) | 4 (57%) | 78 (55%) |
|  | Total | 232 | 79 | 7 | 7 | 142 |

Supplementary Table 7: Tobacco use pattern within three months following COVID-19 infection.

| **S.no** | **Use pattern** | **N (%) Smokeless tobacco** | **N (%) Smoking tobacco** | **Both users** | |
| --- | --- | --- | --- | --- | --- |
|  |  |  |  | **N (Total=1425) (%) Smoking tobacco** | **N (Total=1425) (%) Smokeless tobacco** |
|  | Daily | 193 (13.5%) | 52 (3.6%) | 6 (0.42%) | 7 (0.5%) |
|  | Weekly | 13 (0.9%) | 15 (1%) |  |  |
|  | Monthly | 22 (1.5%) | 3 (0.2%) |  |  |
|  | Never | 4 (0.28%) | 9 (0.6%) | 1 (0.07%) |  |
|  | Total | 232 (16%) | 79 (5.4%) | 7 (0.5%) | 7 (0.5%) |

The pattern of alcohol consumption:

Concerning alcohol users (n=142), the severity pattern among the study population was slightly different from tobacco users. The majority (n=78, 55%) of them had a severe degree of dependence, 40 (28%) of alcohol users were having ‘moderate,’ i.e. hazardous alcohol use, and 24 (17%) were found to have ‘mild,’ i.e. low risk of alcohol use. Of the total number of users of alcohol (n=142), who reported intake of alcohol before getting infected, the majority (n=66, 46.4%) resorted to once weekly alcohol use, followed by daily use (n=43, 30.3%) and once monthly use (n=33, 23.3%).

Supplementary Table 8: Alcohol use pattern among the study population

| **S. No** | **Alcohol Use** | **N (Total=1425) (%)** | |
| --- | --- | --- | --- |
|  | Lifetime use | 142 (9.96%) | |
|  | Using alcohol within 3 months before the onset of COVID-19 | 132 (9.2%) | |
|  | Using alcohol within 3 months following COVID-19 illness | 147 (10.3) | |
| **Gender distribution of lifetime alcohol use (N=142)** | | | |
|  | Female | 21 (15%) | |
|  | Male | 121 (85%) | |
| **Alcohol use pattern before and after COVID-19 infection** | | | |
|  | | **Before** | **After** |
|  | Daily | 43 (3.02) | 46 (3.2) |
|  | Weekly | 66 (4.63) | 66 (4.6) |
|  | Monthly | 33 (2.32) | 35 (2.4) |
|  | Total | 142 (9.96) | 147 (10.3) |

Supplementary Table 9: Comparison of National Family Household Survey-5 (NFHS-5) data with the present study findings.

|  | **NHFS-5** | **Current study** | **P-value** |
| --- | --- | --- | --- |
| **Tobacco use prevalence** | 11.7% (141/1202) | 22% (318/1425) | <0.00001 |
| **Alcohol use prevalence** | 4.8% (58/1202) | 10% (142/1425) | <0.00001 |

### Association between socio-demographic and clinical parameters with COVID-19 disease severity:

The severity of COVID-19 infection was assessed as ‘mild’. ‘moderate’ and ‘severe’ was assessed for having any statistical association with various socio-demographic and clinical parameters of the study population. Severe illness was found to be significantly more (x^2^=66.979, p< 0.0001) in the elderly population (>60 years; 17.72%) followed by those of middle-aged (30-60 years) (4.52%) and young age (12-30 years) (1.59%). A similar age-wise severity pattern was also seen for those with moderate illness severity (14.55% vs 8.46% vs 6.37%). Mild illness was more in the young age group (92.03%). Females in the study population had more mild (86.27% vs 85.52%) and severe (5.58% vs 5.42%) illnesses but lesser of moderate illnesses (8.13% vs 9.04%) than males, and this was not statistically significant (x^2^=0.3135, p=0.85). Those who resided in a rural background had more of mild (87.33% vs 85.31%) and moderate (9.09% vs 8.68%) illness but lesser severe illness (3.57% vs 5.99%) than the urban counterpart. This was also not significantly associated with illness severity (x^2^=2.758, p=0.25). Disease severity, however, was significantly associated with SES of the study population (x^2^=16.302, p<0.0002). Those above the poverty line (APL) (6.74%) had more severe COVID-19 illness than those who were below the poverty line (BPL) (1.87%), while there was a lesser difference between these two SES classes in moderate (9.50% vs 6.70%) and milder (83.74% vs 91.42%) illness. Place of work was also found to be not significantly associated (x^2^=6.3954, p=0.17) with COVID-19 severity. The study included patients from such a time of infection when they could avail of both the two COVID-19 doses, the primary and the booster. At the time of the study, only 5% of the study population received both doses of the vaccination. There was no significant difference in the severity of infection among the two groups (χ² = 1.213, df = 2, p = 0.5452). The presence of comorbidity also showed a significantly higher (x^2^=76.519, p< 0.0001) chance of severe infection than those who reported not having any comorbidity (10.33% vs 2.74%), and also a more chance of moderate infection (14.61% vs 5.48%).

Tobacco use before and within three months of COVID-19 infection was found to be significantly associated (x^2^=6.4766, p=0.03) with moderate severity of the infection in comparison to those who had not used tobacco during that period (12.26% vs 7.76%). This was not with those who reported to have consumed alcohol. All the severity patterns of infection were similar for those who consumed alcohol and those who reported having not consumed it (x^2^=0.0391, p=0.98).

Supplementary Table 10: Association of socio-demographic and clinical variables with Severity

| **S.no** | **Independent variables** | **Severity** | | | **Chi-square** | **Degree of freedom** | **p-value** |
| --- | --- | --- | --- | --- | --- | --- | --- |
|  |  | **Mild** | **Moderate** | **Severe** |  |  |  |
| 1 | **Age:** | 67.72% | 14.55% | 17.72% | 66.979 | 4 | **<0.0001** |
|  | Elderly |  |  |  |  |  |  |
|  | Middle age | 87% | 8.46% | 4.52% |  |  |  |
|  | Young age | 92.03% | 6.37% | 1.59% |  |  |  |
| 2 | **Gender:** | 86.27% | 8.13% | 5.58% | 0.3135 | 2 | 0.85 |
|  | Female |  |  |  |  |  |  |
|  | Male | 85.52% | 9.04% | 5.42% |  |  |  |
| 3 | **Background:** | 85.31% | 8.68% | 5.99% | 2.758 | 2 | 0.25 |
|  | Urban |  |  |  |  |  |  |
|  | Rural | 87.33% | 9.09% | 3.57% |  |  |  |
| 4 | **Socio-economic status:** | 83.74% | 9.50% | 6.74% | 16.302 | 2 | **0.0002** |
|  | Above poverty line |  |  |  |  |  |  |
|  | Below poverty line | 91.42% | 6.70% | 1.87% |  |  |  |
| 5 | **Vaccination status:** | 85.55% | 8.85% | 5.60% | 1.213 | 2 | 0.5452 |
|  | Incomplete vaccination |  |  |  |  |  |  |
|  | Completed vaccination | 89.86% | 7.25% | 2.90% |  |  |  |
| 6 | **Occupation:** | 85.90% | 8.18% | 5.90% | 6.3954 | 4 | 0.17 |
|  | Neither home nor hospital based/related work |  |  |  |  |  |  |
|  | Work from/at home | 82.96% | 13.33% | 3.70% |  |  |  |
|  | Work in/associated with hospital | 87.61% | 9.52% | 2.85% |  |  |  |
| 7 | **Co-morbidity:** | 75.04% | 14.61% | 10.33% | 76.519 | 2 | **<0.0001** |
|  | Yes |  |  |  |  |  |  |
|  | No | 91.77% | 5.48% | 2.74% |  |  |  |
| 8 | **Tobacco use:** | 83.01% | **12.26%** | 4.71% | 6.4766 | 2 | **0.03** |
|  | Yes |  |  |  |  |  |  |
|  | No | 86.54% | **7.76%** | 5.69% |  |  |  |
| 9 | **Alcohol use:** | 85.21% | 9.15% | 5.63% | 0.0391 | 2 | 0.98 |
|  | Yes |  |  |  |  |  |  |
|  | No | 85.81% | 8.72% | 5.45% |  |  |  |

### Association between socio-demographic and clinical parameters with complications of COVID-19:

Complications from COVID-19 infection were mentioned in sections 3.3 and 3.4. Those who were more than 60 years of age had significantly more complications (36.70%) (x^2^=21.516, p<0.0001) than those who were of the age group of 30-60 years (30.31%) and 12-30 years (17.52%). This was also observed in the fact that complications were absent significantly more (x^2^=21.516, p<0.0001) in those above 60 years of age (63.29%) than those who were of 30-60 years (69.68%) and 12-30 years (82.47%). Females, as opposed to males had significantly less post-COVID-19 complications (24.18% vs 30.75%; x^2^=6.003, p=0.014). Complications were reportedly found to be significantly more in those who hailed from an urban background than a rural background (31.24% vs 19.80%; x^2^=14.862, p<0.0001), and also in them who belonged from above poverty level (32.88% vs 17.15%; x^2^=32.49, p<0.0001). Complications were also found to be significantly more felt by those who worked in a hospital or associated with one than those who worked from home and neither of the above (39.04% vs 33.33% vs 27.34%; x^2^=7.962, p=0.018). With regards to vaccination status, it was observed that those who had taken a double dose of the COVID-19 vaccine during their illness had a significantly lesser proportion of complications (13.4%) compared to those who had not completed vaccination (29.7%) (χ² = 7.965, df = 1, p = 0.004769). The association between comorbidities and complications was also studied. Those who reported comorbidities in the study were found to experience significantly more complications than those who reported having no comorbidities (47.75% vs 18.09%; x^2^=139.55, p<0.0001). The association is equally true from a different perspective. Study participants without comorbidities had a significantly higher chance of having no complications than those with comorbidities (81.09% vs 52.24%; x^2^=139.55, p<0.0001).

Concerning tobacco and alcohol use by the study population, significant associations have been found between their consumption/ use and post-COVID complications. Tobacco users had a significantly higher chance of developing complications than non-users (38.99% vs 25.83%; x^2^=20.233, p<0.0001). Similarly, consumers of alcohol also had significantly higher complications than non-consumers (40.14% vs 27.51%; x^2^=9.34, p<0.002).

Supplementary Table 11: Association of various sociodemographic and clinical variables with Complications

| **S.no** | **Independent variables** | **Complications** | | **Chi-square** | **Degree of freedom** | **p-value** |
| --- | --- | --- | --- | --- | --- | --- |
|  |  | Present | Absent |  |  |  |
| 1 | Age: | 36.70% | 63.29% | 21.516 | 2 | **<0.0001** |
|  | Elderly |  |  |  |  |  |
|  | Middle age | 30.31% | 69.68% |  |  |  |
|  | Young age | 17.52% | 82.47% |  |  |  |
| 2 | Gender: | 24.18% | 75.81% | 6.0031 | 1 | 0.014 |
|  | Female |  |  |  |  |  |
|  | Male | 30.75% | 69.24% |  |  |  |
| 3 | Background: | 31.24% | 68.75% | 14.862 | 1 | **<0.0001** |
|  | Urban |  |  |  |  |  |
|  | Rural | 19.80% | 80.19% |  |  |  |
| 4 | Socio-economic status: | 32.88% | 67.11% | 32.49 | 1 | **<0.0001** |
|  | Above poverty line |  |  |  |  |  |
|  | Below poverty line | 17.15% | 82.84% |  |  |  |
| 5 | Vaccination status: | 29.57% | 70.43% | 7.965 | 1 | **0.004769** |
|  | Not completed vaccination |  |  |  |  |  |
|  | Completed vaccination | 13.04% | 86.96% |  |  |  |
| 6 | Occupation: | 27.34% | 72.65% | 7.9632 | 2 | **0.018** |
|  | Neither home nor hospital based/related work |  |  |  |  |  |
|  | Work from/at home | 33.33% | 66.66% |  |  |  |
|  | Work in/associated with hospital | 39.04% | 60.95% |  |  |  |
| 7 | Co-morbidity: | 47.75% | 52.24% | 139.55 | 1 | **<0.0001** |
|  | Yes |  |  |  |  |  |
|  | No | 18.09% | 81.09% |  |  |  |
| 8 | Tobacco use: | 38.99% | 61.00% | 20.233 | 1 | **<0.0001** |
|  | Yes |  |  |  |  |  |
|  | No | 25.83% | 74.16% |  |  |  |
| 9 | Alcohol use: | 40.14% | 59.85% | 9.3404 | 1 | **<0.002** |
|  | Yes |  |  |  |  |  |
|  | No | 27.51% | 72.48% |  |  |  |

### Structural equation modelling:

Multiple iterations were tried to construct a model using latent variables from the manifest variables with severity and complications outcome variables. A significant model could not be obtained. The best model that could be explained from the data is depicted in Figure 3. The model based on latent variables Demographic (Dmg) (Gender (Gnd) and Age (Ag_)), Social Determinants (ScD) (Socio-economic status (SES), Occupation (Occ), and Background (Bck)), modifiable factors (MdF) (Vaccination (Vcc) and Comorbidity (C_M)), and Substance use (Sbs) (Alcohol use (Al_) and Tobacco Use (Tb_)) with the Severity (Svr) and complications (An_) could not achieve a best fit (CFI-0.83, TLI-0.734, RMSEA-0.06, Chi-square <0.05).

Supplementary Figure 2: Structural equation modelling.


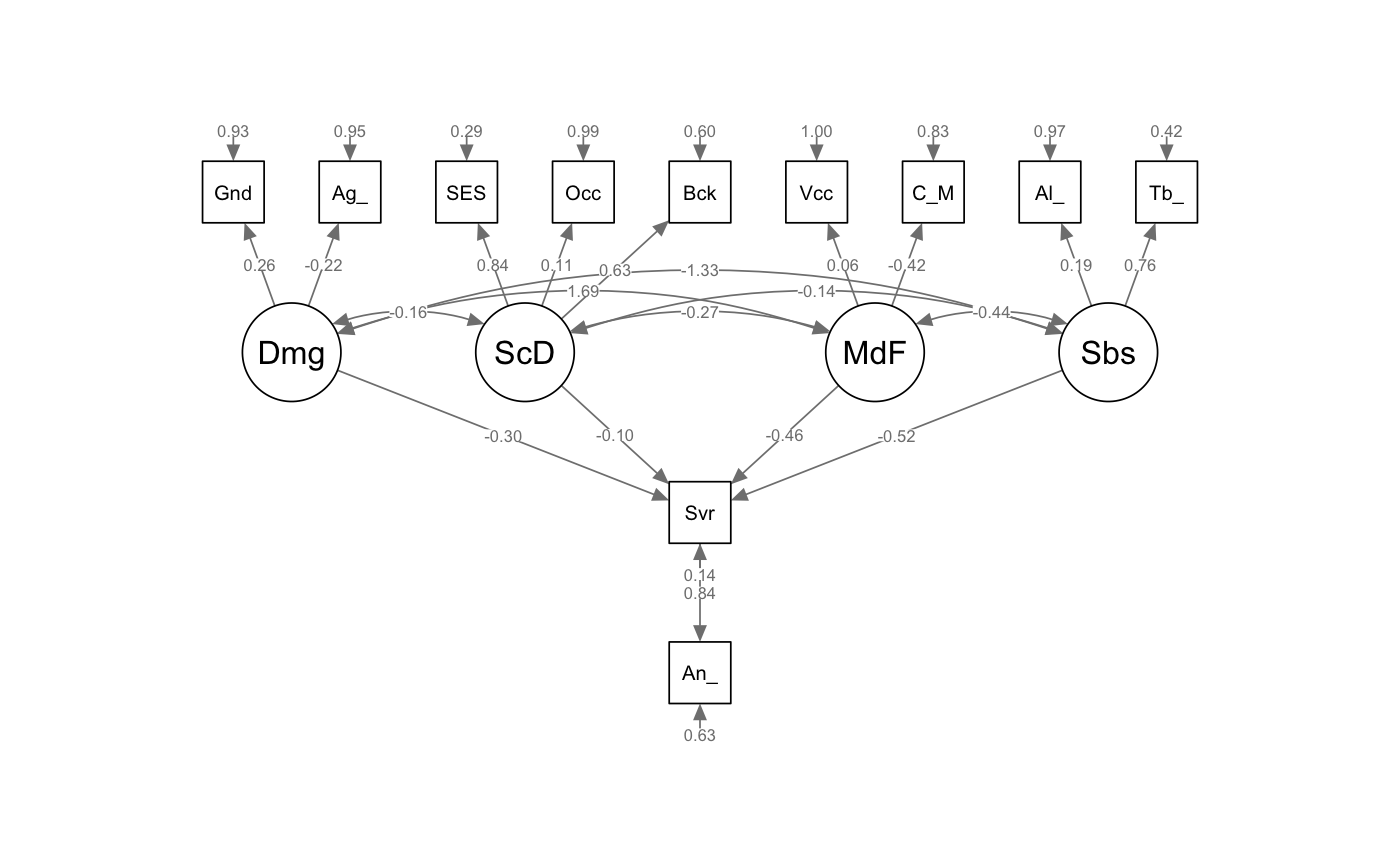


Latent variables: Demographic (Dmg) (Gender (Gnd) and Age (Ag_)), Social Determinants (ScD) (Socio-economic status (SES), Occupation (Occ), and Background (Bck)), modifiable factors (MdF) (Vaccination (Vcc) and Comorbidity (C_M)), and Substance use (Sbs) (Alcohol use (Al_) and Tobacco Use (Tb_)) with the Severity (Svr) and complications (An_)

# **Questionnaire:**

(The research assistant will enter the responses on the JotForm (online survey form) on the tablets).

| Question | Response |
| --- | --- |
| Unique ID number |  |
| Date of COVID-19 infection  (According to the government source) |  |

Respondent detail

| S. No | Question | Response |
| --- | --- | --- |
|  | You are answering the following questions in regard to-  Family member to be interviewed and ticked if the patient died during the COVID-19 pandemic | - Patient - Family member |

Socio-demographic data

| S. No | Question | Response |
| --- | --- | --- |
|  | Gender | - Male - Female - Transgender |
|  | Age in years | - <15 years - 15-30 years - 31-45 years - 46-60 years - >60 years |
|  | Socio-economic status  (According to Jharkhand government) | - BPL card holder - APL card holder |
|  | Occupation (what is the nature of your occupation) | - Work from/at home - Work in/associated with hospital - Neither home nor hospital based/related work |
|  | Background | - Urban - Rural |

COVID-19 related questions:

| S. No | Question | | | | | | Response |
| --- | --- | --- | --- | --- | --- | --- | --- |
|  | At any time in life, have had | | | | | |  |
|  | 1. Diabetes | | | Yes | | No |  |
|  | 1. High blood pressure | | | Yes | | No |  |
|  | 1. Heart disease | | | Yes | | No |  |
|  | 1. Kidney disease | | | Yes | | No |  |
|  | 1. Cancer | | | Yes | | No |  |
|  | 1. Chronic lung disease | | | Yes | | No |  |
|  | 1. Mental illness | | | Yes | | No |  |
|  | 1. Others (any chronic illness) | | | Yes | | No |  |
| 8. | Have you been vaccinated before you were tested positive during the COVID-19? | | | | | | - No - Single dose - Two doses |
| 9. | What happened during COVID-19 illness? | | | | | |  |
|  | 1. Had no symptoms | Yes | | | | No |  |
|  | 1. Had symptoms but did not need hospitalisation | Yes | | | | No |  |
|  | 1. Needed hospitalisation | Yes | | | | No |  |
|  | 1. Needed oxygen | Yes | | | | No |  |
|  | 1. Needed ICU care | Yes | | | | No |  |
|  | 1. Passed away (if about family member) | Yes | | | | No |  |
| 10 | Did you have the any of the following new health problems within three months after recovery from COVID-19? | | | | | |  |
|  | 1. Heart related | | Yes | | No | |  |
|  | 1. Kidney related | | Yes | | No | |  |
|  | 1. Lung related | | Yes | | No | |  |
|  | 1. Blood related | | Yes | | No | |  |
|  | 1. Auto-immune illness | | Yes | | No | |  |
|  | 1. New infections | | Yes | | No | |  |
|  | 1. Neurological problems | | Yes | | No | |  |
|  | 1. Loss of smell | | Yes | | No | |  |
|  | 1. Inability to recognise objects | | Yes | | No | |  |
|  | 1. Psychological problems | | Yes | | No | |  |
|  | 1. Others | | Yes | | No | |  |

Tobacco related questions

| S. No | Question | Response |
| --- | --- | --- |
|  | Anytime in life, have you ever used any of these tobacco products?  (If none, skip tobacco related questions) | - Smoking (such as bidi, cigarette, cigar, etc) - Smokeless (such as gutkha, Khaini, other chewing tobacco) - Both - None |
|  | For how many years have you been using/used these tobacco products? | - Less than 5 years, - 5-10 years - More than 10 years |
|  | In the three months before your COVID infection did you use tobacco products? | - Yes (current use) - No, I was using before that (Ever use) |
|  | In the three months before your COVID infection, how often have you been using these tobacco products: | - Never - Daily - Weekly - Monthly |
|  | After the COVID-19 infection, what has been your pattern of use of tobacco products: | - Never - Daily - Weekly - Monthly |
|  | Fagerstorm – smoking  1. How soon after you wake in the morning do you smoke or first use tobacco?   - Within 5 minutes 3 - 6 to 30 minutes 2 - 31 to 60 minutes 1 - More than 60 minutes 0   2. Do you find it difficult not to use tobacco where tobacco is forbidden?   - Yes 1 - No 0   3. Which of the cigarette would you most hate to give up?   - First thing in the morning 1 - Any other time 0   4. Do you use tobacco when you are sick enough to have to stay in the bed?   - Yes 1 - No 0   5. How many cigarettes do you smoke a day?   - 10 or less 0 - 11-20 1 - 21to 30 2 - 31 or more 3   6. Do you use tobacco more in the morning than the rest of the day?   - Yes 1 - No 0 | Total score |
|  | Fagerstorm smokeless tobacco  1. After a normal sleeping period, do you use  Smokeless within 30 minutes of waking?   - Yes 1 - No 0   2. Do you use smokeless tobacco when you are  sick or have mouth sores?   - Yes 1 - No 0   3. How many times do you use per week?   - Less than 2 times 0 - More than 2 times 1 - More than 4 times 2   4. Do you intentionally swallow your tobacco  juices rather than spit?   - Never 0 - Sometimes 1 - Always 2   5. Do you keep a dip or chew in your mouth  almost all the time?   - Yes 1 - No 0   6. Do you experience strong cravings for a dip or chew when you go for more than two hours  without one?   - Yes 1 - No 0   7. On average, how many minutes do you keep a fresh dip or chew in your mouth?   - 10-19 minutes 1 - 20-30 minutes 2 - More than 30 minutes 3   8. What is the length of your dipping day (total  hours from first dip/chew in a.m. to last dip/chew in p.m.)?   - Less than 14.5 hours 0 - More than 14.5 hours 1 - More than 15 hours 2   9. On average, how may dips/chews do you take each day?   - 1 - 9 times 1 - 10 - 15 times 2 - >15 times 3 | Total score |

Alcohol related questions

| S. No | Question | Response |
| --- | --- | --- |
|  | Did you ever use alcohol (like brandy, whisky, beer or local drinks like toddy, mahua)?  If answer is yes, please apply Alcohol Use Disorder Identification Test (AUDIT)  If no, skip alcohol related questions | - No - Yes, in the last 3 months before I was tested with COVID-19 (current use) - Yes, 3 months earlier to COVID-19 infection (ever use) |
|  | After the COVID-19 infection, what has been your pattern of alcohol use | - Never - Daily - Weekly - Monthly |


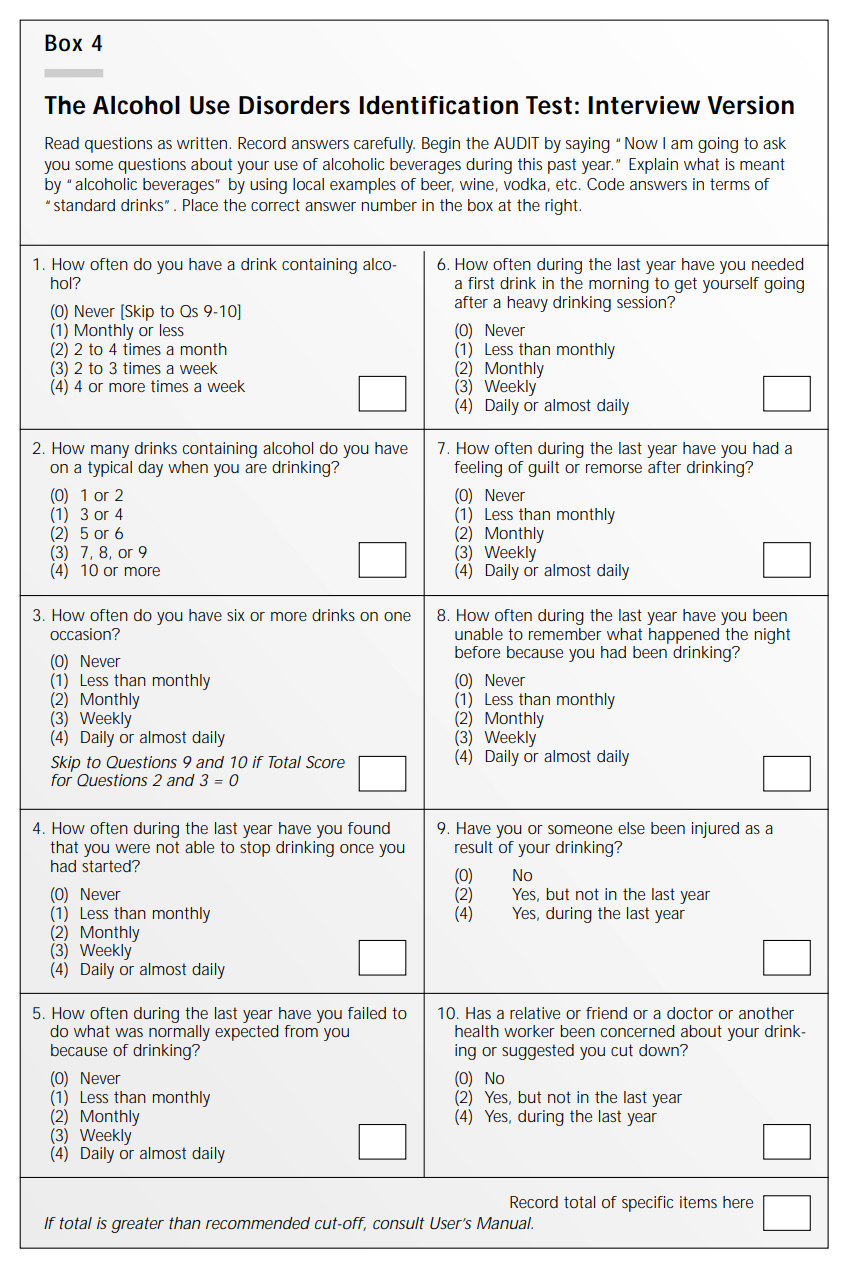


Mental health related questions

| S. No | Question | Response |
| --- | --- | --- |
|  | Were you diagnosed with psychiatric illness in the past like anxiety/depression?  (If yes, please mention the diagnosis) | - Yes - No |
|  | Did you develop any psychological problems after you were diagnosed with COVID-19? (Screening tool to be used) | - Yes - No |

a. Generalized Anxiety Disorder (GAD-2)

| Over the last 2 weeks, how often have you been bothered by the following problems? | Not at all | Several Days | More than half the days | Nearly everyday |
| --- | --- | --- | --- | --- |
| 1. Feeling nervous, anxious, or on edge | 0 | 1 | 2 | 3 |
| 2. Not being able to stop or control worrying | 0 | 1 | 2 | 3 |

b. Patient Health Questionnaire-2 (PHQ-2)

| Over the last 2 weeks, how often have you been bothered by the following problems? | Not at all | Several Days | More than half the days | Nearly everyday |
| --- | --- | --- | --- | --- |
| 1. Little interest or pleasure in doing things | 0 | 1 | 2 | 3 |
| 2. Feeling down, depressed or hopeless | 0 | 1 | 2 | 3 |

c. Primary Care PTSD Screen for DSM-5 (PTSD-5)

| In the past month, have you… | | |
| --- | --- | --- |
| 1. Had nightmares about the event (s) or thought about the event (s) when you did not want to? | Yes | No |
| 1. Tried hard not to think about the event (s) or went out in a way to avoid situations that reminded you of the event (s)?3 | Yes | No |
| 1. Been constantly on the guard, watchful or easily startled? | Yes | No |
| 1. Felt numb or detached from people, activities or your surroundings? | Yes | No |
| 1. Felt guilty or unable to stop blaming yourself of others for the event (s) or any problems the event (s) may have caused? | Yes | No |
| Total score is sum of ‘Yes’ responses in items 1-5 | Total Score |  |

d. Others:

7. Concluding questions

| S. No | Question | Response |
| --- | --- | --- |
|  | Would you like to seek help for your tobacco or alcohol use? (Only to be asked for tobacco and alcohol users)  If yes, link to tobacco Quitline and national Quitline for alcohol | - Yes - No |

####

IEC approval


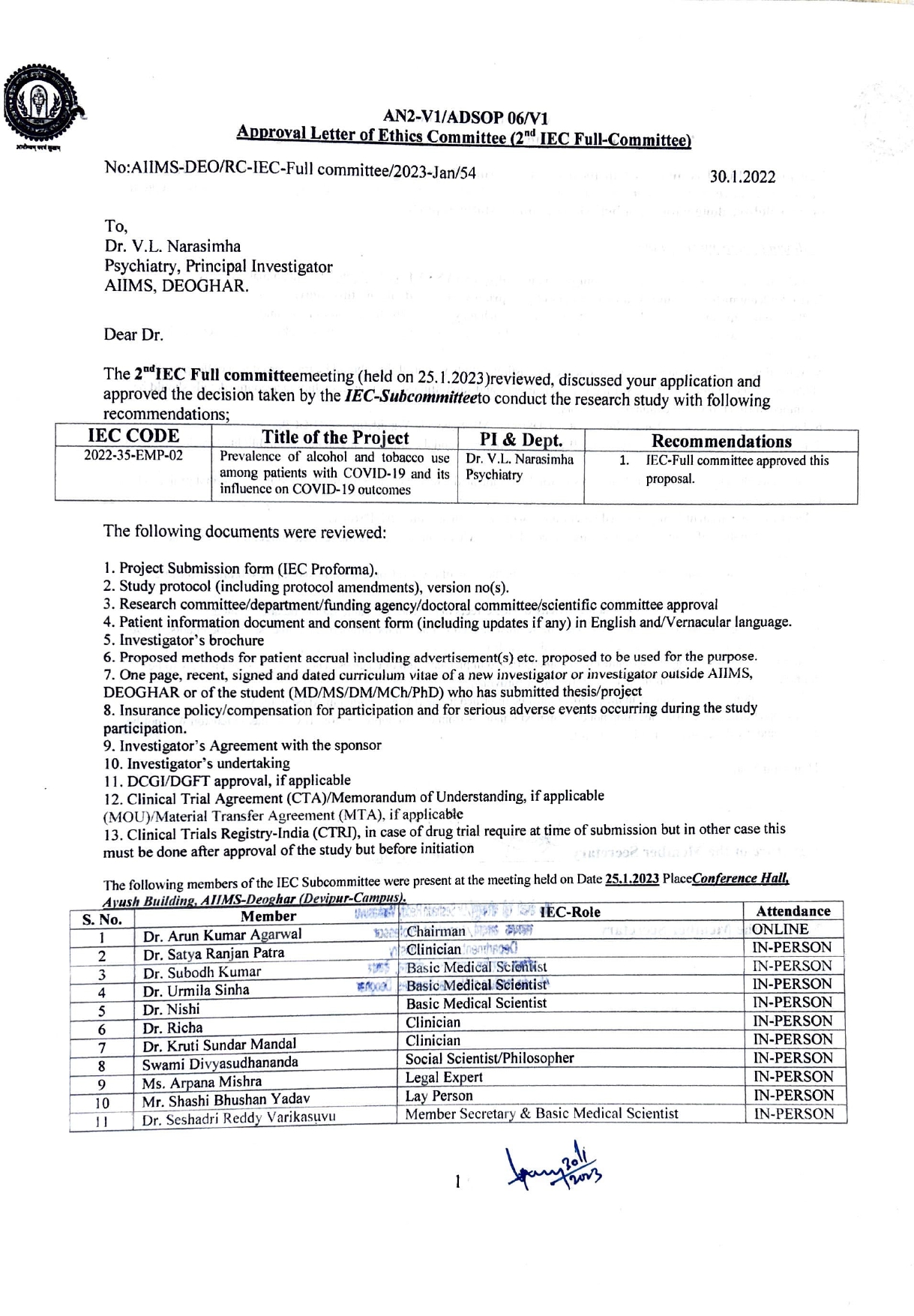

Supplement: Supplementary file 1 [file Data_Sheet_1.docx]
